# Device-Measured Change in Physical Activity in Primary School Children During the UK COVID-19 Pandemic Lockdown: A Longitudinal Study

**DOI:** 10.1123/jpah.2022-0434

**Published:** 2023-05-04

**Authors:** James Scales, Jasmine Chavda, Erika Ikeda, Ivelina Tsocheva, Rosamund E Dove, Helen E Wood, Harpal Kalsi, Grainne Colligan, Lewis Griffiths, Bill Day, Cheryll Crichlow, Amanda Keighley, Monica Fletcher, Chris Newby, Florian Tomini, Fran Balkwill, Borislava Mihaylova, Jonathan Grigg, Sean Beevers, Sandra Eldridge, Aziz Sheikh, James Gauderman, Frank Kelly, Gurch Randhawa, Ian S Mudway, Esther van Sluijs, Christopher J Griffiths

**Affiliations:** 1 Asthma and Lung UK Centre for Applied Research, Edinburgh,United Kingdom; 2 Wolfson Institute of Population Health, Barts and The London School of Medicine and Dentistry, Queen Mary University of London, London,United Kingdom; 3 Institute for Health Research, University of Bedfordshire, Luton,United Kingdom; 4 MRC Epidemiology Unit, University of Cambridge, Cambridge,United Kingdom; 5 Usher Institute, The University of Edinburgh, Edinburgh,United Kingdom; 6 University of Nottingham, Nottingham,United Kingdom; 7 Centre of the Cell, Blizard Institute, Barts and The London School of Medicine and Dentistry, Queen Mary University of London, London,United Kingdom; 8 MRC Centre for Environment and Health, Imperial College London, London,United Kingdom; 9 MRC-Asthma UK Centre in Allergic Mechanisms of Asthma, London,United Kingdom; 10 Department of Population and Public Health Sciences, University of Southern California, Los Angeles, CA,USA

## Abstract

**Background:**

Lockdown measures, including school closures, due to the COVID-19 pandemic have caused widespread disruption to children’s lives. The aim of this study was to explore the impact of a national lockdown on children's physical activity using seasonally-matched accelerometery data.

**Methods:**

Using a pre/post observational design, 179 children aged 8-11 years provided physical activity data measured using hip worn tri-axial accelerometers worn for 5 consecutive days pre-pandemic and during the Jan-Mar 2021 lockdown. Multilevel regression analyses adjusted for covariates were used to assess the impact of lockdown on time spent in sedentary and moderate-to-vigorous physical activity (MVPA).

**Results:**

A 10.8-minute reduction in daily time spent in moderate-to-vigorous physical activity (standard error [SE]: 2.3min/day, P<0.001), and a 33.2-minute increase in daily sedentary activity (SE: 5.5min/day, P<0.001) were observed during lockdown. This reflected a reduction in daily MVPA for those unable to attend school (-13.1±2.3 min/day, P<0,001) during lockdown, with no significant change for those who continued to attend school (0.4±4.0min/day, P<0.925).

**Conclusion:**

These findings suggest that the loss of in-person schooling was the single largest impact on physical activity in this cohort of primary school children in London, Luton and Dunstable UK.

## Introduction

The restrictions placed on populations during the coronavirus pandemic caused unprecedented disruption to the lives of children. Governments worldwide implemented national stay-at-home quarantines (lockdowns) to slow the spread of the virus. In the United Kingdom (UK) multiple regional and national lockdowns were in place for periods between March 2020 and February 2022 (Figure 1), where UK law during national lockdowns stipulated that people could only leave their homes for essential work, food shopping, medical needs and exercise. This also included implementation of “remote” schooling for most children, except for vulnerable children and children whose parents were involved in ‘key worker’ occupations (e.g. emergency services personnel, teachers, and workers involved in the production and sale of food).

Physical activity has been associated with numerous health benefits in children, including a reduction in early-life risk factors for cardiovascular disease and obesity^1,2^,. Pre-lockdown research has suggested that active travel methods of commuting to school contribute to physical activity in children, national lockdowns have impacted this method of physical activity. Moreover, pre-pandemic research assessing the structured-day hypothesis has long held that schools are an important source of structure and routine, which instigates and encourages physical activity for children beyond simply the active travel component ^3,4^. School closures, alongside the closure of other sports facilities, such as sports centres, sports clubs, play areas and swimming pools, further limited the opportunities to engage in physical activity during periods of lockdown.

A substantial body of research has already documented some of the effects of COVID-19 lockdowns on children’s perceived physical activity levels. These studies are summarised in recent systematic and narrative reviews ^5–7^. Broadly, children’s physical activity is reported to decline and sedentary activity increase during a lockdown, and this has been consistent regardless of life stage and country of residence. Specifically, engagement with physical activity has been shown to be dependent on sociodemographic factors. Findings from an ethnically-diverse cohort in England, UK showed that White British children were more ‘sufficiently active’ (34.1%) compared to children of Pakistani heritage (22.8%), or children of ‘any other ethnic group’ (22.8%)^8^. A survey of 1214 children in Ireland also highlighted that the lack of in-person schooling limited physical activity by reducing active travel^9^. However, to date no research has employed device-assessed methods to explore the mediating effects of ethnicity or loss of active travel on lockdown physical activity.

While self-report questionnaires facilitate large sample sizes and are easy to complete with participants confined to their homes, they are commonly associated with many disadvantages such as recall bias ^10^ and mood-congruence bias ^11^. Few studies have reported on device-measured physical activity data during lockdowns. One study among Dutch primary school children used accelerometery data from 66 children (10.5±3.6 years), reporting that sedentary time was increased by 45 minutes per day and total physical activity was 17 minutes per day lower while attending school under national lockdown ^12^. A longitudinal study conducted with 800 children, aged 8 to 18, in Wales showed that moderate-to-vigorous physical activity (MVPA) measured by accelerometery increased by 38.4 minutes per day when children returned to school after a stay-at-home lockdown ^13^. Finally, a repeated measures cross-sectional design has highlighted that children engaged in 7 minutes per day less MVPA while attending school during the pandemic ^14^. To date, research has only reported total daily physical activity, and not considered physical activity during school time, meaning it has not been possible to ascertain the specific contribution of school attendance compared to out-of-school physical activity to total physical activity levels. Understanding the specific role of school-based physical activity is critical to informing future school-based initiatives.

While a few studies have reported physical activity change during general national lockdowns, no previous studies have quantified the change in accelerometer-derived physical activity in UK children before and during a national lockdown with school closures. Therefore, the primary aim of this study was to determine the impacts of the third national UK lockdown (06/01/2021-08/03/2021) on daily and school time MVPA and sedentary activity, using seasonally-matched accelerometery data from primary school children during school closures in England, UK. Secondary aims were to explore potential moderators of change in MVPA and self-reported physical activity. We hypothesised that children’s physical activity would be lower during school closures in lockdowns.

## Methods

### Participants

Participants were recruited from the Children’s Health in London and Luton (CHILL) study. CHILL is a multi-ethnic cohort recruited in 2018-2019 from 84 schools in Central London, Luton and Dunstable to evaluate the impact of London’s Ultra-Low Emission Zone (ULEZ) on health. To be included in CHILL, children had to be attending primary schools located in Luton and Dunstable or within or very close to the border of London’s ULEZ. Further details about the cohort recruitment can be found elsewhere ^15^. Participants in the CHILL study were sent invites to participate in this sub-study in January 2021, while aged between 8-11 years.

In the first instance, our contacts at the primary schools were approached by email to gain assent to approach participants' parents/guardians. Invitations to participate were then sent to participants' parents by SMS. Parents were offered the opportunity to ask any questions via phone or email. Assent was obtained from nominated parents or guardians to collect data during lockdown. Written consent was provided by participants’ parents during recruitment to the CHILL study. All procedures were performed in accordance with the declaration of Helsinki and the institutional research ethics committee provided research approval (QMERC: 2018/08).

### Study timeline

Time spent in MVPA and sedentary activity is known to be influenced by seasonal variation. To minimise the impact of seasonal variation on the current analyses ^16^, participants had to have provided acceptable accelerometer wear between the dates of 01/11/2019 and 15/03/2020, as baseline (pre-lockdown) comparison to match follow-up data collection (06/01/2021-08/03/2021).

The follow-up data collection time point (06/01/2021-08/03/2021) occurred during the third UK national lockdown (Figure 1). During these dates, the UK was under strict stay-at-home lockdown where schools offered online learning to students, with the exception of vulnerable children, or the children of ‘key workers’, who were permitted to attend school in person.

### Participant demographics and mode of transport

Child’s ethnicity and mode of travel to school were reported by parents on a baseline questionnaire. Participants’ parents and participants were individually asked “During a normal week, how often does your child/do you travel to school using the following modes of transport”, with the option to respond, “Never”, “1-2 days”, “3-4days”, “Everyday” for the modes of “Walk”, “Scooter”, “Bike”, “Private car”, “Taxi”, “Bus”, Train/tube”. To support the final analysis the transport modes were condensed; walk, scooter and bike were categorised as active travel, while train/tube and bus were categorised as public transport, and taxi and private car were categorised as private transport. Mode of transport was presented as the method which was used for the most days per week once all scores had been summed.

In the first instance a parents score to the question was reported as the outcome variable, however if a parent reported a score that classified a participant as using two modes of transport, or a spurious result, the child’s responses to “How did you travel to school today?”, were also included to the calculation to provide a single transport method.

### Device-measured physical activity

Actigraph GT3X+ (Actigraph Corp, USA) tri-axial accelerometers were used to collect physical activity data. The children wore the accelerometer on their right hip using an elasticated belt. Valid daily wear time at baseline was set at five consecutive days of 480 minutes (eight hours) between 6am and 11pm. This threshold was chosen in agreement with previous large cohort research ^17–19^. Baseline data was collected during routine research visits to schools as part of the CHILL study. Participants were provided with an accelerometer and instruction sheet by researchers who explained that they should be worn at all times except when sleeping, swimming, or showering. Participants were instructed to wear the accelerometer for seven days, then return it to the school, after which a researcher collected them. Accelerometers were initialised to start recording at 9am on the day they were handed out.

Due to restrictions during UK lockdown, all follow-up measures took place remotely. An accelerometer, an instruction sheet were mailed to the participant’s home address with a self-addressed, freepost envelope for return.

Accelerometers were initialised to start collecting two days after they had been placed in the post. Participants were asked to start wearing the accelerometer as soon as it arrived, regardless of whether it was a weekday or weekend, and to wear it for five days before returning via self-addressed, prepaid envelope.

Accelerometer data were downloaded and exported at five-second intervals using the ActiLife software (Version 6.13.4). Data were processed through ActiLife software to mark periods of 60 minutes or greater of continuous zero (tri-axial) counts as non-wear and apply thresholds to calculate time spent at different intensity levels Data files with hourly-level data were then subsequently processed in Stata (Version 13, StataCorp. College Station, TX, USA) to remove periods of 60 minutes or more of continuous zero acceleration. Cut-points defined by Evenson et al., ^20^ were applied to classify sedentary activity(<101 counts per minute) and MVPA (≥ 2296 counts per minute) using uniaxial data. School time MVPA and sedentary activity were defined as any activity at the required threshold between 9am-3pm on weekdays. Accelerometers were set up to only collect data for seven days.

Anthropometric data (measured by trained researchers and reported as age- and sex-specific body mass index), ethnicity and mode of travel were collected during baseline as part of the CHILL study health assessments and data collection methods are described elsewhere^20^.

### Data analysis

To account for variance in wear time across data collection waves, three-level multilevel mixed effects models were used to analyse the accelerometer data (see supplementary material 1for details on data structure). Ten different multilevel mixed effects models were run. Four were used to assess the primary aim exploring changes in MVPA during lockdown compared to baseline. School time model 1 and total daily model 1 included key demographic data as covariates (wave, age at baseline, wear time, BMI, Chill study site, gender, ethnicity and whether children attended school during lockdown). While total daily models 2, 3 and 4 kept the same demographic input but included ethnicity (Model 2), school attendance (Model 3) or transport to school (Model 4) as effect modifiers (full model descriptions are available in supplementary material 2). The same design was used to examine sedentary behaviour with Total Daily Model 5 including data as covariates only, and Models 6, 7 and 8 including modifiers for ethnicity, school attendance and transport to school, respectively. The ethnicity variable in Total daily models 2 and 6 was converted to dummy variables except the ‘Other’ category. The same design was used to examine sedentary activity with Model 5 including data as covariates only, and Models 6, 7 and 8 including modifiers for ethnicity, school attendance and transport to school, respectively. MVPA and sedentary activity during school time were assessed via multilevel models with the same demographic inputs and no moderating variables as a sensitivity analysis.

P-values ≤ 0.05 were considered statistically significant for all statistical tests. All analysis was conducted using STATA MP (Version 13, StataCorp. College Station, TX, USA).

## Results

Out of a cohort of 3414 CHILL participants, 802 children provided valid baseline accelerometery data between the pre-defined matched baseline dates. Due to a limited number of accelerometers available, a random sample of 633 (79%) of these children were sent an SMS message inviting them to take part in the study, and 233 (37%) consented to taking part. 192 (82%) returned accelerometers, of whom 179 (93%) participants wore them and provided acceptable data for analysis. 38 (21%) children attended school at least one day during national lockdown while wearing an activity monitor. Table 1presents descriptive baseline characteristics of the 179 participants with acceptable accelerometery data.

Valid total wear time at baseline was 749.89 (116.88) min/day and 733.37 (117.14) min/day during lockdown. Multilevel mixed effects models for school time (School time model) and daily MVPA (Totally daily models 1,2,3,4) exploring the impact of lockdown are summarised in Table 2with more detailed models presented in supplementary material 2.

The school time model shows a significant reduction of time spent in MVPA during lockdown compared to baseline (-10.8±1.8min/day, P<0.001). Total daily model 1 presents a significant reduction of time spent in MVPA during lockdown compared to baseline (-10.8±2.3min/day, P<0.001). When effect modifiers were included in subsequent models, no significant interaction effect was observed for ethnicity (x^2^ (4)= 3.33, P=0.505), or mode of travel to school (x^2^ (2)= 1.58, P=0.4530). Significant interaction was observed for school attendance, which was characterised by a statistically significant reduction in daily MVPA for those not able to attend in-person schooling (-13.1±2.3 min/day, P<0,001) during lockdown and no change for those attending school (0.4±4.0min/day, P<0.925) during lockdown.

Multilevel mixed effects models for school time (School time model) and daily sedentary activity (Total daily models 5,6,7,8) exploring the impact of lockdown are summarised in table 3with further detail presented in supplementary material 3.

The school time model (Table 3) presents a significant increase in time spent in sedentary activity during lockdown compared to baseline (28.6±4.3min/day, P<0.001). The total daily model (Model 5), presents a significant increase in daily sedentary activity during lockdown compared to baseline (32.2±5.5 min/day, P<0.001). When effect modifiers were included in subsequent models, no interaction effect for ethnicity (x^2^ (4)=2.14, P=0.711), or mode of travel (x^2^(2)=1.39, P=0.500) was observed. A significant interaction was observed for school attendance, which was characterised by a significant increase in sedentary activity for those unable to attend school in person during lockdown (39.6±5.6min/day, P<0.0001) and no change in sedentary activity for those attending school during lockdown (2.2±9.9min/day, P=0.822).

## Discussion

The aim of this study was to determine the impact of a UK lockdown on primary school children’s daily physical activity in and outside of school time using accelerometery. This is amongst the first studies to report device-based measures of physical activity during a lockdown with school closures. Sedentary activity increased while MVPA decreased during lockdown in comparison to before lockdown measures. Critically, this change was almost completely attenuated in children who attended school in person during the lockdown. This was supported by significant reductions in physical activity during school time among those not attending school. These findings suggest that the removal of in-person schooling and not the closure of sports clubs was likely the largest impact on physical activity in this cohort.

This study observed that children’s total daily MVPA was reduced by an average of 10.8 minutes compared to before lockdown baseline. Our findings are comparable to a Dutch study which saw a reduction of 17.0 minutes of daily MVPA during lockdown with school attendance and another UK study which saw an increase of 12.4 minutes of daily MVPA as children returned to school following school closure lockdown ^13^ and a US study which observed reductions of 7 minutes during lockdown ^21^. The reduced difference between Dutch and US cohorts and our UK cohort is notable considering the Dutch and US children were attending school during lockdown data collection. The different data collection and analysis techniques may partially account for this difference. However, large observational studies have shown that country-specific lockdown guidelines may account for different levels of physical activity ^22^.

The present study observed significant increases of 33.2 min/day of sedentary activity, broadly in line with the Dutch (45 minute increase) and US cohorts (145 minute increase) while a UK cohort saw a reduction of 90 minutes in sedentary time when children returned to school after a stay-at-home lockdown^13^. Possible contributing factors for the large differences seen between sedentary activity include the use of different thresholds to define sedentary activity, differences in the cohorts studied, i.e. older children, and the lack of before-pandemic baseline data for comparison. However, in general this shift from MVPA to sedentary activity is consistent in direction with large UK survey-based research ^8,23^.

Such findings are concerning as it is well known that physical activity habits are formed in childhood ^24–26^. Scientific consensus shows that physical activity habit forming in childhood is critical to preventing obesity and chronic illness, and maximising health prospects ^1,27^. Lockdown restrictions, and thus physical activity restrictions, during this critically formative time may therefore increase the risk of chronic health problems later in life.

Pre-lockdown research has suggested that ethnicity may predict engagement with physical activity, with research suggesting that South East Asian primary school-aged children may be less likely to engage in physical activity ^28^. Moreover, national self-reported UK data has highlighted that Black children were less likely than Asian and White children to meet Government daily physical activity guidelines of 60 minutes MVPA, during lockdown compared to before lockdown baseline ^8^. When using device-measured physical activity, the present study found that the impact of the lockdown did not differ by ethnicity. Notably, self-reported data in a predominantly South East Asian UK inner-city cohort broadly comparable to the present sample, suggested that children of Pakistani heritage were less likely to meet the same Government physical activity guidelines^28^. However, the researchers noted that no difference occurred when trips outside the home were added as a confounder, highlighting that parental and demographic variability may have a greater impact than ethnicity itself.

Interestingly, school attendance (children who were permitted to go to school during lockdown as their parents were classified as ‘key workers’) highly predicted change in MVPA and sedentary activity. Children who attended school maintained pre-pandemic levels of MVPA and sedentary activity, showing that school attendance plays a key role in maintaining physical activity. This suggests schools were unable to encourage physical activity by remote learning methods during stay-at-home lockdowns. This is in agreement with post-lockdown work which shows that when attending in-person schooling were more physically active compared to virtual-schooling at home^29^.

The observed moderating effect of school attendance is further supported by a reduction in MVPA of a similar magnitude during school time. It has been postulated that young children are able to mitigate against periods of sedentary activity by intuitively engaging in informal play forms of physical activity ^30^. It is likely that children have been able to compensate for closures of sport clubs and general restrictions to movements outside of the home that have occurred outside of school time, but not loss of physical activity associated with school activities. This novel finding suggests that school closures were potentially the primary source of shift from MVPA to sedentary activity in children during lockdown.

It is long established that vulnerable groups such as those from deprived backgrounds, low income households, or in poor health are less likely to engage in physical activity^28,31^. Schools mitigate health inequalities by providing access to welfare and wellbeing resources and support. It is likely that school closures will negatively impact vulnerable groups, further increasing health inequality.

There are two commonly proposed methods of how school attendance encourages healthy behaviours, such as increased physical activity. Firstly, pre-pandemic work has long suggested that school attendance increases physical activity by increasing active travel forms of commuting to the location^26^, providing structure to engage in physical activity via physical education lessons, break times and at after school clubs ^32^ and through unstructured play during break and lunch times. Secondly, the structured day hypothesis postulates that school attendance provides vital daily routine which promotes healthy behaviours such as physical activity. The sudden closure of schools during lockdown has enabled a natural quasi experiment of the structured day hypothesis. This study observed no differences children who travelled to school by active and non-active travel methods. However, when combined with significant drops in school time physical activity, it can be suggested that the daily structure provided by school attendance itself is significant in mediating the observed decrease in physical activity, providing support for the structured day hypothesis and suggesting that active travel to school has only a small impact on physical activity.

Children engage in physical activity in many settings. Most environments involve familial engagement, such as attending sports clubs and swimming pools, informal play in parks with friends and family, and active methods of commuting and transportation. Pre-lockdown research has shown that schools provide a uniquely egalitarian environment to encourage physical activity predominantly through structured physical education lessons ^32^ and unstructured play during break and lunch times ^33^. Thus, these findings suggest school closures during such critically formative life stage may increase health inequalities, by disproportionately impacting the most vulnerable children in society.

It is notable that schools were unable to promote physical activity during remote learning. While national lockdowns propose a unique challenge, long term absence from school is a common and persistent challenge for many children. The findings of this work suggest that providing daily structure may encourage physical activity during absence from school.

The findings of this study suggest that a strong argument can be made for providing schools with further resources to support children in returning to pre-lockdown levels of physical activity. Additionally, resources should be developed to help children engage in physical activity during periods of long-term school absence. It is encouraging that previous research has observed a reduction of 90 minutes in daily sedentary time after returning to school, which highlights some rebound in physical activity following the easing of lockdown restrictions, however a sequential cross-sectional design has shown that children have not returned to pre-pandemic levels of physical activity^14^ Future research could explore whether children returned to pre-pandemic baseline levels of physical activity.

Methodological strengths of this study include the use of a repeated measures study design and device-measured physical activity at seasonally-matched time points before and during lockdown with school closure. Despite this, the limitations of this work should be acknowledged: This study used a convenience sample from an ongoing study (CHILL Study) which recruited inner-city children. While the CHILL cohort is ethnically representative of Central London and Luton, this population might not be fully representative of the UK population as a whole.

The seasonally-matched within-participant design is a strength, allowing comparison of measures collected before and during a national lockdown. Previous large observational research has shown that total physical activity in children reduces by 4.2% annually, characterised predominantly by a shift from light physical activity to increased sedentary activity. This suggests that sedentary changes at follow up could have been overestimated ^34^. However, it should be noted that no change in physical activity occurred in children attending school suggesting in this cohort there is a reduced risk of overestimation. Moreover, retrospective baseline measures from the parental questionnaires may be subject to recall or selection bias.

Our findings show that children have been able to mitigate the impact of closures of sports clubs and after school activities; however, they experienced significant drops in physical activity during school time. Schools play a critical role in encouraging physical activity and this importance is only increased during lockdown. We have observed that schools were unable to remotely support children in engaging in physical activity during stay-at-home lockdowns. This loss in school-based physical activity at such a critical habit-forming life stage may impact children’s long-term development and increase health inequalities by impacting children with less out-of-school support to engage in physical activity. It is not clear whether children return to baseline levels of physical activity; previous work has shown that physical activity does increase following the removal of lockdown^19^, however, it is not yet clear whether this means a return to baseline levels of activity.

## Supplementary Material

Supplementary Material S1

Supplementary Material S2

Supplementary Material S3

## Figures and Tables

**Figure:**
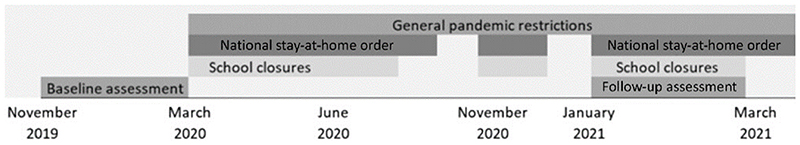


**Table 1:** Baseline (before-lockdown) demographic profile of study sample

Variable	Descriptive (n)	Cohort distribution (%)
Site		
London	122	68.2
Luton	57	31.8
Gender		
Male	70	39.11
Female	109	60.89
Ethnicity		
Asian	41	23.70
Black	29	16.76
Mixed	22	12.72
White	69	39.88
Other	12	6.94
Mode of travel to school		
Active travel	102	56.98
Private transport	46	25.70
Public transport	31	17.32
Age, (years: mean, SD)	8.83 (0.75)	
BMI (z-score: mean, SD)	0.15 (1.13)	
Total Sedentary time (min/day)	530.62 (100.75)	
Total MVPA(min/day)	55.75 (24.86)	

Age, BMI and physical activity variables presented as mean with SD in parenthesis

**Table 2:** Summary of results of hierarchical linear modelling for time spent in MVPA

Parameter	School time model	Total daily model 1	Total daily model 2	Total daily model 3	Total daily model 4
Regression coefficients					
Intercept	24.5 (9.9), P=0.014	49.0 (13.5), P<0.001	48.3 (14.7), P<0.001	44.0 (13.6), P=0.001	47.4 (13.7), P=0.001
Lockdown	-10.8 (1.8), P<0.001	-10.8 (2.3), P<0.001	-15.6 (6.6), P=0.017	0.4 (4.0), P=0.925	-10.0 (2.6), P<0.001
Age	-0.5 (1.0), P<0.637	-2.0 (1.5), P=0.172	-2.0 (1.5), P=0.168	-2.0 (1.5), P=0.172	-1.9 (1.5), P=0.197
Wear time	0.1 (0.0), P<0.001	0.1 (0.0), P<0.001	0.1 (0.0), P<0.001	0.1 (0.0), P<0.001	0.1 (0.0), P<0.001
School attendance at lockdown (attend/none)	-7.4 (2.1), P<0.001	-5.0 (2.9), P=0.088	-5.0 (2.9), P=0.089	1.1 (3.5), P=0.748	-5.00 (2.9), P=0.09
Site (London/Luton)	-2.8 (1.6), P<0.083	-5.0 (2.3), P=0.031	-4.9 (2.3), P=0.032	-5.0 (2.3), P=0.028	-4.9 (2.6), P=0.061
Gender (Male/Female)	-8.7 (1.6), P<0.001	-11.5 (2.3), P<0.001	-11.5 (2.3), P<0.001	-11.6 (2.3), P<0.001	-11.4 (2.3), P<0.001
Ethnicity group (White)			0.4 (5.7), P=0.945		
Asian	-5.8 (2.0), P<0.003	-7.1 (2.8), P<0.011	-5.6 (5.9), P=0.342	-7.2 (2.8), P=0.010	-7.2 (2.8), P=0.010
Black	-4.1 (2.3), P<0.073	-8.1 (3.3), P<0.013	-5.6 (6.2), P=0.365	-8.2 (3.3), P=0.013	-8.8 (3.4), P=0.010
Mixed	-7.6 (2.4), P<0.002	-7.4 (3.5), P<0.032	-8.9 (6.3), P=0.162	-7.4 (3.4), P=0.032	-7.8 (3.5), P=0.025
Other	-2.9 (3.4), P<0.388	-3.2 (4.8), P<0.503		-3.1 (4.8), P=0.519	-3.4 (4.8), P=0.484
BMI-for-age	0.0 (0.7), P<0.952	-1.2 (1.0), P<0.249	-1.2 (1.0), P=0.250	-1.2 (1.0), P=0.237	-1.1 (1.0), P=0.259
Private transport					1.1 (3.3), P=0.750
Public transport					4.9 (3.9), P=0.215
Effect modifiers					
Lockdown x White			6.1 (6.8), P=0.370		
Lockdown x Black			1.4 (7.4), P=0.845		
Lockdown x Asian			3.6 (7.1), P=0.608		
Lockdown x Mixed			10.0 (7.6), P=0.188		
Lockdown x Absent				-13.5 (4.0), P=0.001	
Lockdown x Private transport					-0.3 (3.7), P=0.937
Lockdown x Public transport					-5.5 (4.5), P=0.216

**Table 3:** Summary of results of hierarchical linear modelling for objective physical activity measured by sedentary behaviour

Parameter	School time model 2	Total daily model 5	Total daily model 6	Total daily model 7	Total daily model 8
Regression coefficients					
Intercept	-76 (22.8), P=0.001	-174.0 (31.2), P<0.001	-165.1 (34.0), P<0.001	160.2 (31.3), P=0.001	-169.4 (31.5), P<0.001
Lockdown	28.6 (4.3), P<0.001	32.2 (5.5), P<0.001	36.4 (16.4), P=0.027	2.2 (9.9), P=0.822	30.0 (6.3), P<0.001
Age	5.1 (2.4), P=0.031	11.3 (3.4), P=0.001	11.3 (3.4), P<0.001	11.3 (3.4), p<0.001	11.1 (3.4), P=0.001
Wear time	0.7 (0.0), P<0.001	0.8 (0.0), P<0.001	0.8 (0.0), P<0.001	0.8 (0.0), P=0.001	0.8 (0.0), P<0.001
School attendance at lockdown (attend/none)	21.6 (4.7), P<0.001	19.0 (6.8), P=0.005	19.0 (6.8), P=0.005	1.7 (8.2), P=0.837	18.9 (6.8), P=0.005
Site (London/Luton)	1.9 (3.7), P=0.600	7.1 (5.3), P=0.179	7.1 (5.3), P=0.183	7.3 (5.3), P=0.169	7.3 (6.0), P=0.227
Gender (Male/Female)	8.8 (3.6), P=0.016	13.0 (5.2), P=0.013	12.9 (5.20, P=0.013	13.0 (5.2), P=0.012	12.6 (5.2), P=0.015
Ethnicity group (White)			-6.8 (13.5), P=0.618		
Asian	11.2 (4.5), P=0.013	9.8 (6.5), P=0.129	-0.2 (14.0), P=0.990	10.0 (6.5), P=0.123	10.0 (6.5), P=0.122
Black	12.3 (5.3), P=0.019	23.9 (7.6), P=0.002	9.4 (14.7), P=0.521	23.9 (7.6), P=0.002	25.6 (7.8), P=0.001
Mixed	10.5 (5.6), P=0.058	11.6 (7.9), P=0.144	2.6 (15.1), P=0.863	11.6 (7.9), P=0.145	12.5 (8.0), P=0.117
Other	10.5 (7.8), P=0.175	10.9 (11.0), P=0.319		10.7 (10.9), P=0.329	11.4 (11.0), P=0.300
BMI-for-age	-0.6 (1.6), P=0.692	2.0 (2.3), P=0.379	2.0 (2.3), P=0.380	2.1 (2.3), P=0.365	1.9 (2.3), P=0.397
Private transport					-5.7 (7.9), P=0.472
Public transport					-12.2 (9.3), P=0.190
Effect modifiers					
Lockdown x White			-8.9 (17.2), P=0.605		
Lockdown x Black			7.6 (18.6), P=0.684		
Lockdown x Asian			-2.0 (18.7), P=0.910		
Lockdown x Mixed			-4.1 (19.1), P=0.829		
Lockdown x Absent				37.4 (10.0), P<0.001	
Lockdown x Private transport					6.1 (9.3), P=0.514
Lockdown x Public transport					12.5 (11.3), P=0.267

Parameter estimate presented with standard error in parenthesis. Outcome reported in minutes.
