# Supplementary material for: Device-Measured Change in Physical Activity in Primary School Children During the UK COVID-19 Pandemic Lockdown: A Longitudinal Study
Source: J Phys Act Health. Author manuscript; Available in PMC 2023 Aug 9. (PMC7614900; doi:10.1123/jpah.2022-0434)
Supplement: Supplementary Material S1 [file EMS181945-supplement-Supplementary_Material_S1.pdf]

Supplementary Material S1: Data Structure

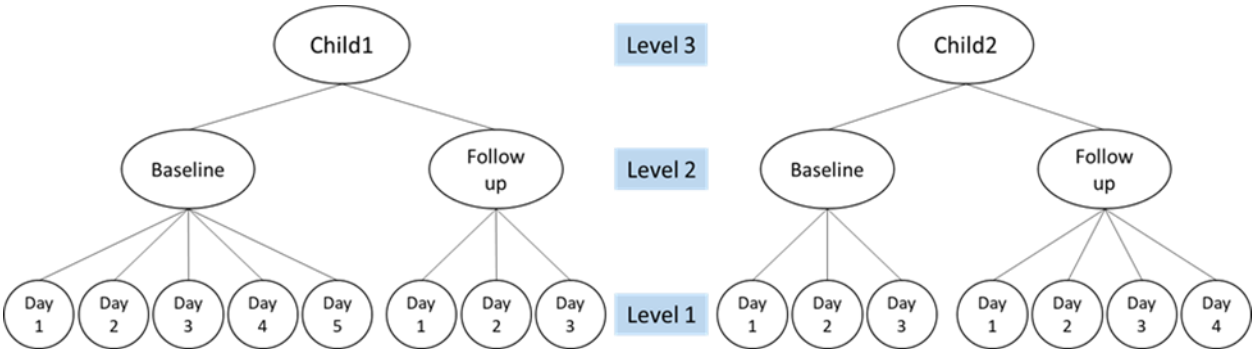

Level 3 ID	Level 2 ID	Level 1 ID	Response	Level 1 covariates	Level 2 covariates*	Level 3 covariates
k	j	i	y_{ijk}	x_{1ijk}	x_{2jk}	x_{3k}
Child	Wave	day	MVPA	Age	Wave	Attended school
				Weartime	-	CHILL Study Site
						Gender
						Ethnicity
						Age specific BMI
						Travel mode (Models 4 and 9)

*The effects of any wave-level covariates in this model cannot be estimated as the wave dummy (0/1) have destroyed all wave-level variation.**For Analysis of sedentary data, Response will be replaced with sedentary (defined by Evenson et al 2008)
